# The Effect of Noise Trauma and Deep Brain Stimulation of the Medial Geniculate Body on Tissue Activity in the Auditory Pathway

**DOI:** 10.3390/brainsci12081099

**Published:** 2022-08-18

**Authors:** Faris Almasabi, Gusta van Zwieten, Faisal Alosaimi, Jasper V. Smit, Yasin Temel, Marcus L. F. Janssen, Ali Jahanshahi

**Affiliations:** 1 Department of Neurosurgery, Maastricht University Medical Center, 6229 HX Maastricht, The Netherlands; 2 School for Mental Health and Neuroscience, Faculty of Health, Medicine and Life Sciences, Maastricht University, 6229 ER Maastricht, The Netherlands; 3 Department of Physiology, Faculty of Medicine, King Khalid University, Abha 62529, Saudi Arabia; 4 Department of Physiology, Faculty of Medicine, King Abdulaziz University, Rabigh 25732, Saudi Arabia; 5 Department of Ear Nose and Throat, Head and Neck Surgery, Zuyderland, 6419 PC Heerlen, The Netherlands; 6 Department of Clinical Neurophysiology, Maastricht University Medical Center, 6229 HX Maastricht, The Netherlands

**Keywords:** tinnitus, deep brain stimulation, medial geniculate body

## Abstract

Tinnitus is defined as the phantom perception of sound. To date, there is no curative treatment, and contemporary treatments have failed to show beneficial outcomes. Deep brain stimulation has been suggested as a potential therapy for refractory tinnitus. However, the optimal target and stimulation regimens remain to be defined. Herein, we investigated metabolic and neuronal activity changes using cytochrome C oxidase histochemistry and c-Fos immunohistochemistry in a noise trauma-induced rat model of tinnitus. We also assessed changes in neuronal activity following medial geniculate body (MGB) high-frequency stimulation (HFS). Metabolic activity was reduced in the primary auditory cortex, MGB and CA1 region of the hippocampus in noise-exposed rats. Additionally, c-Fos expression was increased in the primary auditory cortex of those animals. Furthermore, MGB-HFS enhanced c-Fos expression in the thalamic reticular nucleus. We concluded that noise trauma alters tissue activity in multiple brain areas including the auditory and limbic regions. MGB-HFS resulted in higher neuronal activity in the thalamic reticular nucleus. Given the prominent role of the auditory thalamus in tinnitus, these data provide more rationales towards targeting the MGB with HFS as a symptom management tool in tinnitus.

## 1. Introduction

Tinnitus is an auditory perception in the absence of an actual external sound. The prevalence of tinnitus is approximately 15%, and has a significant impact on the quality of life of approximately 1.2% of the general population [1]. Tinnitus patients frequently have comorbid depression, anxiety and sleep disorders [2]. Given the lack of effective treatment options, the use of both non-invasive and invasive neuromodulation approaches have been the subject of investigation during the last decade [3,4]. Deep brain stimulation (DBS) is an invasive neuromodulatory procedure successfully applied in several neurological disorders [5]. In animal studies, positive effects of DBS on tinnitus-like behaviour have been reported in different targets in the auditory pathway, including the medial geniculate body (MGB), the inferior colliculus (IC) and the dorsal cochlear nucleus (DCN) [6,7,8,9]. Other brain targets have also been suggested to alleviate tinnitus, including areas within the limbic system, basal ganglia and cerebellum [4]. However, the optimal DBS target for effective treatment of tinnitus remains to be determined. The role of the auditory thalamus, particularly the MGB, in tinnitus pathology is also attracting more interest [10]. Findings in both human and animal studies suggest the auditory thalamus has a prominent role in the altered auditory system in tinnitus [11,12]. Increased spontaneous firing rate in the MGB and decreased functional connectivity between the MGB and auditory cortices are reported in tinnitus [12]. Moreover, the MGB is indirectly influenced by limbic regions via the thalamic reticular nucleus (TRN). From a neurosurgical point of view, the MGB is also of interest, as it is accessible stereotactically [13]. In a previous study, we showed that MGB-HFS alleviates tinnitus-like behaviour in rats [9].

Herein, we investigated the effects of noise trauma and MGB-HFS in cortical and subcortical regions involved in tinnitus using a dual approach. Activity markers were used to map the central changes in auditory and limbic areas, whereas motor areas were used as control regions. Cytochrome C oxidase (COX), a neural energy demand marker, was used to assess the metabolic activity in those areas. Additionally, the activity level in the auditory pathway was assessed using c-Fos [14,15]. The network effects of MGB-HFS were investigated using c-Fos immunohistochemistry, which could shed light on the mechanisms of action of DBS.

## 2. Materials and Methods

### 2.1. Study Design and Animals

Rat brain tissues were obtained from two previous studies. In Study 1, the activity changes in a noise trauma-induced rat model of tinnitus were assessed. Animals were divided into a unilateral noise trauma group (1A, n = 9) and a non-exposed control group (1B, n = 5). Single unit and local field potential electrophysiological acquisitions were conducted [16] before the animals were euthanized and tissue collected for post-mortem analysis.

In Study 2, eleven rats were exposed to unilateral noise trauma, and DBS electrodes were implanted bilaterally in the MGB. Rats were assigned to two groups before they were euthanized. The first group (Study 2A, n = 6) was stimulated before euthanasia, and the second group (2B, n = 5) received sham stimulation [9].

In both experiments, adult (10–12 weeks) male Sprague Dawley rats (Charles River, Sulzfeld, Germany) were used. The rats were individually housed in standard Makrolon™ cages and were given ad libitum access to food and water. Their weight at the time of surgery ranged between 250 g and 350 g. The room where the rats were housed was air-ventilated with reversed light/dark cycles (12/12 h), consistent temperature (20 °C to 22 °C) and humidity between 60% and 70%. All experiments were conducted at the Central Animal Facility at Maastricht University during the daytime (dark cycle). The Animal Experiments and Ethics Committee of Maastricht University reviewed and approved all animal procedures.

### 2.2. Noise Trauma

The rats in both studies were exposed to unilateral 16 kHz octave-band noise at 115 dB sound pressure level (SPL) via a speaker (Ultrasonic power amplifier and Ultrasonic Dynamic Speaker Vifa (Avisoft Bioacoustics, Berlin, Germany)) for 90 min under general anaesthesia. The rats were anesthetized with xylazine and ketamine (10 mg/kg, 90 mg/kg, respectively) i.p., and anaesthesia was maintained with ketamine (60 mg/kg/h). The contralateral ear was protected by clay to prevent total hearing loss. The rats were given 3 weeks to recover. The rats in the control group (1B) were only kept under anaesthesia for 90 min and were not exposed to noise trauma.

### 2.3. Gap Prepulse Inhibition of Acoustic Startles

The presence of tinnitus was assessed using the Gap Prepulse Inhibition in Acoustic Startle response test (GPIAS; for a detailed description, see [7]). GPIAS is used for tinnitus screening in rats using the gap detection reflex that was first described by Turner and colleagues [17].

Briefly, rats were placed inside an acoustic cylindrical chamber with a polyethylene floor and vertical bars (diameter 17 cm, height 40 cm). A piezo sensor was attached beneath the chamber to measure the startle force. Auditory stimuli were presented and amplified via the speaker, which was placed in the centre of the ceiling of the acoustic chamber. Sounds were calibrated with a Bruel & Kjaer 2231 dB meter with a 4191 microphone. The rats were able to move freely and were monitored with an infrared camera.

A narrow-band noise was generated at 10 kHz, 16 kHz and 20 kHz at 75 dB SPL background noise. The startle stimulus was a broadband noise lasting 20 ms with a 115 dB peak equivalent SPL. In all gap trials, a silent gap of 50 ms was embedded in the background noise 100 ms prior to the startle stimulus. Each session consisted of alternated 10 gap trials and 10 startle-only trials for every background sound. Prior to that, the rats were allowed 5 min of adaptation and then were habituated by presenting 10 startle-only trials.

The gap/no-gap ratios were calculated as the amplitude of each gap-startle divided by the corresponding mean of no-gap startles. On two consecutive days, each rat received two complete sessions for each condition, with one habituation session at the beginning of the experiment.

The results of these behavioural assessments have been previously published [9,16]. In summary, noise trauma increased the gap/no-gap ratios in the 1A, 2A and 2B groups compared to the 1B group and the baseline measurements in Study 1 and Study 2. These results suggest a successful induction of tinnitus, consistent with previous studies [7,8]. However, confounding effects of hearing loss and hyperacusis cannot be ruled out. In Study 2, HFS resulted in decreased gap/no-gap ratios to the baseline level, which suggests tinnitus reduction during HFS [9].

### 2.4. Surgical Procedures

Study 1: Under general anaesthesia with urethane (1.5 g/kg, i.p.), the rats were mounted on a stereotactic frame (model 51950, Stoelting Co., Wood Dale, IL, USA) with hollow ear bars (World Precision Instruments, Sarasota, FL, USA) to allow the passage of acoustic stimuli. Body temperature was maintained with a heating pad (ATC1000, World Precision Instruments, Sarasota, FL, USA) at 37 °C.

To conduct electrophysiological recordings in the MGB, a small craniotomy contralateral to the noise trauma side was performed at identified coordinates (AP −5.4 mm to −5.8 mm, ML 3.4 mm to 3.5 mm and DV 5.8 mm to 6.8 mm) [18]. After the electrophysiological recordings, the rats were euthanized (for details, see the section on tissue collection).

Study 2: Electrode implantation was performed as described previously [9]. DBS electrodes (coaxial gold-coated with platinum-iridium inner wire, tip diameter of approximately 50 μm, shaft diameter of 250 μm) were implanted in the bilateral MGB (AP −5.7 mm, ML +/− 3.9 mm and DV −6 mm).

### 2.5. Deep Brain Stimulation

In Study 2, DBS was applied using the stimulation settings determined in our earlier study [9]. HFS consisted of monophasic, square-wave pulses with a frequency of 100 Hz, a pulse duration of 60 µs and an amplitude of 100 µA using a self-designed experimental DBS construct. The bipolar electrode was composed of an inner (cathode) and outer (anode) contact (see [19]). The stimulation electrode was connected to a constant-current isolator via a cable (DLS 100; WPI, Berlin, Germany), which was connected to a stimulator (DS8000; WPI, Berlin, Germany). Before euthanasia, group 2A was stimulated with HFS for 1 h, while in group 2B, the electrode was connected to the cable without electrical stimulation for 1 h. Thereafter, all rats were allowed to rest in their cages for 90 min before euthanasia.

### 2.6. Tissue Collection

Study 1: The rats were euthanized under anaesthesia by decapitation, and the brains were quickly removed and frozen in 2-methyl-butane (isopentane) and stored in −80 °C until use. The brains were cut serially in a cryostat (Leica CM3050S, Nussloch, Germany) into 50 μm thick coronal sections.

Study 2: At the end of the experiment, the rats were injected with pentobarbital (120 mg/kg to 180 mg/kg, i.p.). Transcardial perfusion was applied using Tyrode’s buffer (0.1 M), followed by a fixative containing 4% paraformaldehyde, 15% picric acid and 0.05% glutaraldehyde in 0.1 M phosphate buffer (pH 7.6) at 4 °C. The brains were removed and placed in paraformaldehyde overnight at 4 °C and then transferred to 1% sodium azide at 4 °C for long-term storage. The brains were embedded in 10% gelatin (Sigma-Aldrich, Zwijndrecht, The Netherlands) and cut serially on a vibratome (Leica^®^, Wetzlar, Germany) into 30 μm thick coronal sections.

### 2.7. Histochemistry and Immunohistochemistry

#### 2.7.1. Cytochrome C Oxidase Histochemistry

Fresh frozen slides were air dried for 30 min and then immersed in an incubation medium for 35 min at 37 °C on a shaker. The incubation medium consisted of 0.1 M HEPES buffer (175 mL), COX (45 mg), 3.30-diaminobenzidine (DAB; 230 mg), sucrose (9 g) and 1% ammonium nickel sulphate (25 mL) per 200 mL. The slide holder contained an equal number of slides from each group to ensure the observed difference was related to the experimental factor (noise trauma). The slides were removed from the incubation medium before they reached the plateau of the staining intensity (35 min) and transferred to a 4% neutral buffered paraformaldehyde medium for 10 min to stop the reaction. The slides were then dehydrated and coverslipped with Entellen.

Photomicrographs were taken with an AX-70 microscope equipped with a motorized condenser (Olympus, Zoeterwoude, The Netherlands) and guided by Cell-P software (Olympus Soft Imaging Solutions, Münster, Germany) using 4× objective. Regions of interest were delineated in ImageJ (ImageJ software version 1.52p; NIH, Bethesda, MD, USA), and the optical density (OD) was measured. The average OD of multiple sections (two to four based on area size; see Table 1) per rat for each region in both the ipsilateral and contralateral hemispheres was calculated.

COX activity was measured in 16 selected brain areas, including the primary auditory cortex (PAC), the MGB, the IC, the DCN, the basolateral amygdala, the CA1 and CA3 regions of the hippocampus, the lateral habenula (LH), the TRN, the locus coeruleus (LC), the subthalamic nucleus (STN), the substantia nigra (SN), the paraflocculus of the cerebellum, the somatosensory cortex and the dorsal raphe nucleus (DRN), and the posterior commissure was used as a control in the white matter region. All Bregma levels for each region are provided in Table 1 [18].

#### 2.7.2. C-Fos Immunohistochemistry

Sections were incubated for three and two nights in Study 1 and Study 2, respectively, with anti-c-Fos primary antibody (1:2000; rabbit polyclonal; Abcam ab209794). After washing with Tris-buffered saline (TBS) and TBS-Triton X-100 (TBS-T), sections were incubated with secondary antibody (1:800, donkey anti-rabbit biotin; Jackson Immunoresearch Laboratories Inc., Westgrove, PA, USA) for 1 h. This was followed by repeated washing and incubation with avidin–biotin peroxidase complex (1:800, Elite ABC kit, Vectastain, Burlingame, CA, USA) for 2 h. The DAB combined with NiCl2 intensification was used to visualize the staining, and then dehydrated and coverslipped with Pertex (Histolab Products ab, Göteborg, Sweden).

Photomicrographs were also taken with Cell P software (Olympus Soft Imaging Solutions, Münster, Germany) using an AX-70 microscope (Olympus, Zoeterwoude, The Netherlands) with 10× magnification. Cells immunopositive for c-Fos were counted manually after reducing the background by an appropriate threshold level, and then divided by the surface area of interest (cells/mm^2^). In Study 1, the classical auditory regions were analysed, including PAC, the medial part of MGB and the central part of IC and DCN. For Study 2, c-Fos expression was investigated in PAC, the central part of the IC, CA1 of the hippocampus, the dentate gyrus, the basolateral amygdala, the TRN and the DCN.

### 2.8. Statistical Analysis

Sections containing damaged tissue (i.e., cryodamage) were excluded from the analysis. One subject was removed from group 1B due to poor staining quality. A two-way analysis of variance (2-ANOVA) was used to assess the overall changes in c-Fos and COX. The two factors in Study 1 were noise trauma (exposed/non-exposed) and the side of the brain (ipsilateral/contralateral to the noise-exposed ear). In Study 2, the parameters were the stimulation (ON/OFF) and the side of the brain (ipsilateral/contralateral to the noise-exposed ear). For brain regions in the midsagittal line, where no side factor could be tested, a one-way ANOVA test was used. To test the laterality effect of noise trauma, an additional analysis with a t-test was conducted for the significant regions found in the 2-ANOVA. All analyses were performed using SPSS statistical software (SPSS version 25.0, IBM, Chicago, IL, USA). Data were presented as the mean ± standard error of means, and *p*-values < 0.05 were considered significant.

## 3. Results

### 3.1. Cytochrome C Oxidase Density

Based on the noise trauma factor, we found a significant increase in the mean OD (reflecting reduced COX activity) in the noise-exposed group (1A) in the PAC (F(1) = 4.570, *p* = 0.045), MGB (F(1) = 6.292, *p* = 0.021) and CA1 of the hippocampus (F(1) = 11.930, *p* = 0.003). No significant effects were observed in the other areas. Table 1 summarizes the mean OD + (SEM), the Bregma level for each measurement and the statistical outcomes for all regions based on the noise trauma factor.

**Figure 1:**
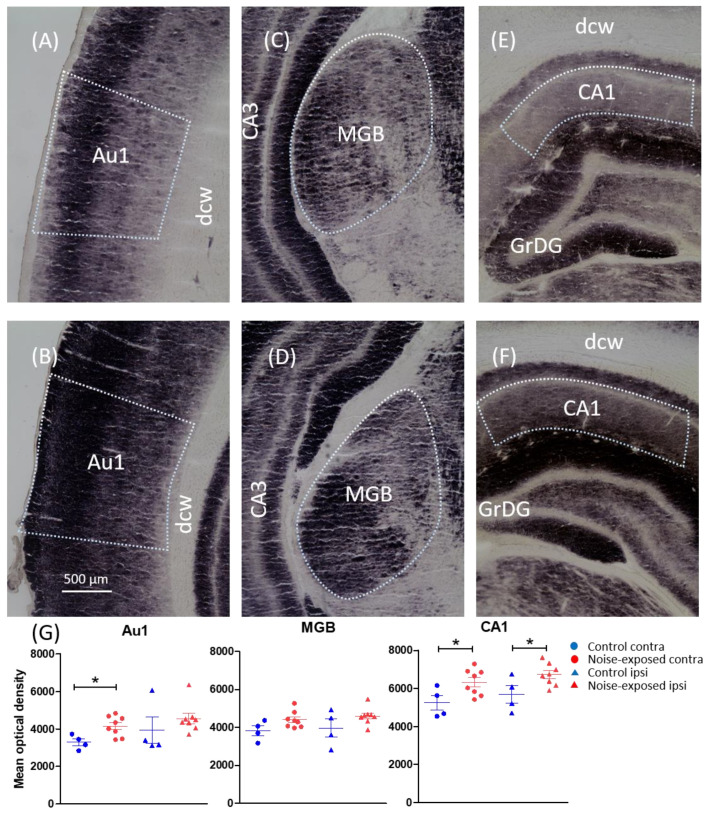
Representative low-power photomicrographs taken from coronal rat brain sections stained with COX. (**A**,**B**) auditory cortex; (**C**,**D**) medial geniculate body; (**E**,**F**) CA1 of the hippocampus. The upper and lower panels represent a noise-exposed and control animal, respectively. (**G**) Graphs show the mean optical density of regions that showed significant changes based on group factors (noise-exposed vs. control). *p* value < 0.05 was defined as significant level and is indicated by an (*). Note that only contralateral Au1 and bilateral CA1 showed significant changes. Scale bar = 500 µm. Au1, primary auditory cortex; dcw, deep cerebral white matter; GrDG, granular layer of dentate gyrus; MGB, medial geniculate body.

Regarding the laterality effect of noise trauma, the PAC was significant only on the contralateral side ((contralateral noise-exposed (N) = 4157, control (C) = 3297, *p* = 0.02), (ipsilateral N = 4563, C = 3941, *p* = 0.34)); changes in contralateral MGB showed a trend, but did not reach statistical significance ((contralateral N = 4405, C = 3829, *p* = 0.07), (ipsilateral N = 4600, C = 3965, *p* = 0.14)); and the CA1 was significant bilaterally ((contralateral N = 6325, C = 5252, *p* = 0.03), (ipsilateral N = 6727, C = 5690, *p* = 0.04)). Figure 1 shows representative photos of COX staining and a graph of the laterality effect of noise trauma.

### 3.2. C-Fos Cell Count

In Study 1, a significant increase in c-Fos expression was found in the 1A group in the PAC region (F(1) = 11.78, *p* = 0.003). No significant changes were observed in the other areas (see Table 2). Regarding the laterality effect of noise trauma, the PAC was significant bilaterally ((contralateral N = 333, C = 189, *p* = 0.046), (ipsilateral N = 299, C = 186, *p* = 0.024)).

**Figure 2:**
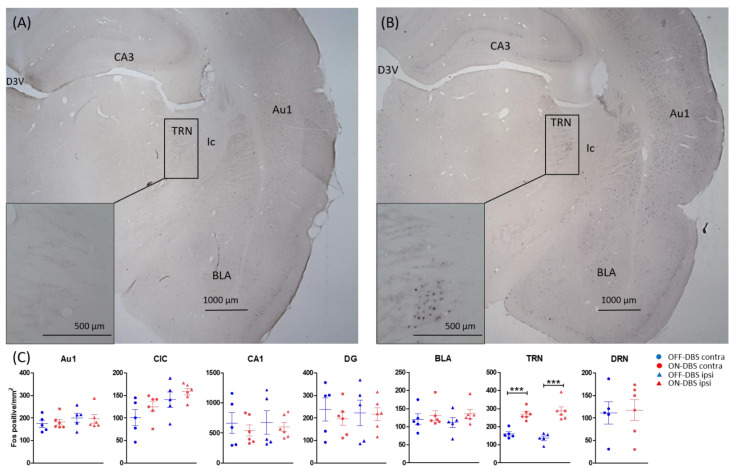
Representative photomicrographs were taken of rat brain sections containing the thalamic reticular nucleus (TRN) in OFF DBS and ON DBS conditions (**A**,**B**, respectively). The sections were immunohistochemically stained using an antibody raised against c-Fos. (**C**) The graphs show the cumulative data of the means and SEMs of c-Fos-positive cells in ON DBS and OFF DBS groups on the ipsilateral and contralateral sides to noise trauma. Note that only TRN showed a significant increase in c-Fos expression in the ON DBS group. *p* values < 0.05 were defined as significant, and *** represents *p* ≤ 0.001. Scale bar = 1000 and 500 in the overview and zoomed box, respectively. Abbreviations: Au1, primary auditory cortex; BLA, basolateral amygdala; CIC, the central part of inferior colliculus; D3V, dorsal third ventricle; DG, dentate gyrus; DRN, dorsal raphe nucleus; Ic, internal capsule; TRN, thalamic reticular nucleus.

In Study 2, a significant increase in c-Fos expression in the TRN was found in the 2A group (F (1) = 65.38, *p* < 0.001) (see Table 3 and Figure 2)). No significant effects were observed in the other areas. Regarding the laterality effect of MGB-HFS stimulation, TRN c-Fos expression was increased bilaterally ((contralateral ON = 270, OFF = 160, *p* < 0.001), (ipsilateral ON = 288, OFF = 136, *p* < 0.001)).

## 4. Discussion

The effects of noise trauma on COX and c-Fos expression levels in cortical and subcortical regions were studied. Metabolic activity, as measured by COX, was reduced in the contralateral PAC and bilaterally in the CA1 of the hippocampus in the noise trauma group. We also observed a trend in the reduction of COX activity in the contralateral MGB (*p* = 0.07). Neuronal activity, assessed by c-Fos expression, was increased bilaterally in the PAC after noise trauma. Assessing the effect of MGB-HFS on neuronal activity revealed increased c-Fos-positive cells in the TRN bilaterally.

### 4.1. Effect of Noise Trauma on Activity Markers

Unilateral noise trauma has been shown to induce unilateral hearing loss in animals, which may also lead to tinnitus-like behaviour. Hyperacusis can also occur [20]. The noise-induced animal model mimics the human aetiology of tinnitus, as hearing loss is the most common cause of chronic tinnitus in humans [21]. However, it is important to acknowledge the presence of hearing loss and possibly hyperacusis when interpreting the findings in this study.

Both auditory and limbic regions are involved in tinnitus pathophysiology [22,23]. Stimulation of some areas within the basal ganglia has been shown to be effective in managing tinnitus symptoms [24,25,26,27,28]. However, the link between these regions and tinnitus has yet to be elucidated. Here, we utilized two widely used activity markers, COX and c-Fos, to explore which brain regions are affected following noise trauma, reflecting the network effects of MGB-HFS. Neither marker distinguishes between specific inhibitory or excitatory neural populations, but they reveal the overall activity changes in the investigated areas. Changes in the activity markers were observed in the PAC, MGB and CA1 of the hippocampus following noise trauma. The involvement of these regions in tinnitus pathophysiology is in line with the findings of several previous studies, at both structural and functional levels [29,30,31,32]. Increased spontaneous firing rate, neuronal synchrony and increased gamma rhythm in the PAC have been frequently reported [33,34,35,36]. The MGB and hippocampus have recently attracted considerable attention and seem to play a key role in tinnitus pathophysiology based on “thalamocortical dysrhythmia” and “thalamic gating” theories [37,38]. Some earlier studies showed an increased spontaneous firing rate and burst activity in the MGB [39,40], while others failed to detect such effects [41]. Furthermore, tinnitus loudness was positively correlated with changes in regional connectivity in the thalamus and hippocampus bilaterally [42]. The hippocampus has been shown to expresses a higher level of Arc protein after acute noise trauma, which is critical to long-term potentiation and depression of synaptic transmission, and thus memory formation [43]. Specifically, reduced GABAergic synaptic densities and cholinergic inputs to the hippocampus were observed in noise-exposed animals that developed tinnitus-like behaviour compared to the controls [44,45].

In our study, the COX expression (i.e., metabolic activity) was surprisingly decreased in all examined regions in noise-trauma animals. To the best of our knowledge, previous studies have not assessed the metabolic activity in tinnitus animals using COX, although our findings are in line with fMRI studies, as the fMRI BOLD signal reflects metabolic activity [46,47]. In a study on a noise trauma-induced model of tinnitus, mice showed a reduced resting-state fMRI signal in the auditory cortex and hippocampus 4 weeks after the noise trauma [48]. Human fMRI studies have reported either reduction in MGB activity or no difference when accounting for the confounder of hyperacusis [12]. The sound-evoked BOLD signal was reported to decrease in the auditory cortex, MGB and hippocampus in tinnitus patients with mild hearing loss and without hyperacusis [49]. Moreover, tinnitus patients with normal hearing levels exhibited a reduced resting-state signal bilaterally in the thalamus [50]. Nevertheless, fMRI outcomes have been contradictory, as several studies have reported an increase [51,52,53,54] or no change in the BOLD signal [55]; hence, it is hard to draw a solid conclusion [32].

Interestingly, we found an increased number of c-Fos-positive cells in the PAC of the noise-exposed group. In previous studies, only a consistent increase in c-Fos expression in the auditory cortex of experimental tinnitus models was reported [56,57,58,59,60]. This is in line with our findings, as well as electrophysiological evidence that shows an increased spontaneous firing rate of the PAC cells in tinnitus-affected animals [60,61].

The increased c-Fos expression and decreased COX activity seem to be contradictory. However, we have had similar observations in DBS studies, where an increased neuronal activity was accompanied by decreased metabolic activity [62]. A possible explanation could be that the increased c-Fos expression or firing rate might not correlate with gross regional metabolic activity.

### 4.2. Effect of the High-Frequency Stimulation in the Medial Geniculate Body on Neuronal Activity Markers

We have previously shown that MGB-HFS alleviates tinnitus-like behaviour [9], yet the mechanism of action remains to be elucidated [12]. Our c-Fos analysis showed a significant increase in the number of c-Fos-positive cells in the TRN in the stimulated group. No significant changes were observed in the PAC, the central part of the IC, the DCN, the CA1 region of the hippocampus, the dentate gyrus or the dorsal raphe nucleus. The TRN is a thin thalamic layer of GABA-ergic neurons that encapsulate the dorsal and lateral parts of the thalamus [63]. It can control the flow of thalamocortical information by its inhibitory feedback to the MGB. It has been suggested that this feedback loop can gate the auditory information and therefore the tinnitus signal at the MGB level [37].

The TRN could play a role in gating of tinnitus signals in several possible ways. Activation of the TRN can attenuate the spontaneous firing rate of neurons in both MGB and PAC neurons [64]. Therefore, TRN activation could suppress the increased firing rate observed in both structures in tinnitus. Furthermore, the TRN plays an important role in regulating thalamocortical oscillations and is considered to be a pacemaker for thalamic firing [65]. Thus, TRN activation following MGB-HFS could plausibly reverse key pathological changes reported in tinnitus.

For the animals whose brains were used in this study, the behavioural assessment of tinnitus was conducted at a group level. Although the noise trauma group showed overall behavioural signs of tinnitus, the presence of tinnitus was not determined in every animal. Therefore, we attribute these results to noise trauma rather than to tinnitus itself. Another potential limitation of this study is that a recording electrode was inserted into the MGB in Study 1, which could induce a lesion effect. Nevertheless, our study results are most likely due to noise trauma, as the insertion was similar in both experimental groups.

## 5. Conclusions

Our results demonstrate that several areas within the auditory pathway (the PAC and MGB) and limbic system (the CA1 of the hippocampus) are affected in the noise trauma-induced rat model of tinnitus. We have shown that MGB-HFS alleviates tinnitus-like behaviour in rats. Examining the tissue of the rats revealed that MGB-HFS increased neuronal activity in the TRN. The effect of MGB-HFS on tinnitus-like behaviour might therefore be mediated by the role of the TRN in the auditory circuit.

## Figures and Tables

**Table 1:** Cytochrome c Oxidase optical density results of the measured regions in noise-exposed group (N) vs. control (C) group.

Region	Bregma	Optical Density	Statistical Outcome
Primary auditory cortex	−3.60, −4.08, −4.56 and −5.04	C: 3619 (361) N: 4360 (171)	F(1, 20) = 4.570, *p* = 0.045
Medial geniculate body	−5.04, −5.52, −6.00 and −6.48	C: 3897 (250) N: 4503 (110)	F(1, 20) = 6.292, *p* = 0.021
Inferior colliculus	−8.04, −8.52 and −9.00	C: 3895 (356) N: 3756 (277)	F(1, 20) = 0.082, *p* = 0.778
Dorsal cochlear nucleus	−10.68, −11.16 and −11.64	C: 3328 (249) N: 3774 (132)	F(1, 20) = 2.946, *p* = 0.102
CA1 of the hippocampus	−3.60, −4.56, −5.52 and −6.36	C: 5471 (288) N: 6503 (172)	F(1, 20) = 10.73, *p* = 0.004
CA3 of the hippocampus	−3.60, −4.56, −5.52 and −6.36	C: 6640 (489) N: 7703 (276)	F(1, 20) = 3.952, *p* = 0.061
Thalamic reticular nucleus	−3.60 and −4.08	C: 5068 (163) N: 5375 (193)	F(1, 20) = 0.974, *p* = 0.336
Basolateral amygdala	−3.60 and −4.08	C: 1987 (368) N: 2196 (323)	F(1, 20) = 0.181, *p* = 0.675
Paraflocculus of the cerebellum	−10.68, −11.16 and −11.64	C: 2722 (199) N: 2905 (133)	F(1, 20) = 0.658, *p* = 0.427
Subthalamic nucleus	−3.60 and −4.08	C: 3114 (249) N: 3141 (125)	F(1, 20) = 0.012, *p* = 0.915
Locus coeruleus	−9.60 and −10.08	C: 4899 (279) N: 4716 (222)	F(1, 20) = 0.230, *p* = 0.637
Lateral habenula	−3.60 and −4.08	C: 3688 (239) N: 4328 (255)	F(1, 20) = 2.321, *p* = 0.143
Substantia nigra	−5.52, −6.00 and −6.48	C: 5439 (114) N: 5450 (152)	F(1, 20) = 0.002, *p* = 0.962
Dorsal-CA3	−5.04 and −6.00	C: 4665 (320) N: 5233 (190)	F(1, 20) = 2.489, *p* = 0.130
Ventral-CA3	−5.04 and −6.00	C: 4042 (188) N: 4519 (171)	F(1, 20) = 2.911, *p* = 0.103
Dorsal-CA1	−5.04 and −6.00	C: 7578 (389) N: 8517 (234)	F(1, 20) = 5.629, *p* = 0.028
Ventral-CA1	−5.04 and −6.00	C: 6521 (502) N: 7298 (284)	F(1, 20) = 2.872, *p* = 0.106
Primary somatosensory cortex	−4.08 and −4.56	C: 3436 (471) N: 3991 (158)	F(1, 20) = 1.790, *p* = 0.196
Dorsal raphe nucleus	−7.20, −7.68 and −8.16	C: 3793 (762) N: 2995 (351)	F(1, 20) = 1.204, *p* = 0.301
Posterior commissure	−4.56 and −5.04	C: 7808 (52) N: 7869 (110)	F(1, 20) = 0.142, *p* = 0.714

The optical density presented as the mean result of each group in both ipsilateral and contralateral regions ± the SEM. The Bregma level where the readout is taken is mentioned as well.

**Table 2:** C-Fos results of the measured regions in noise-exposed group (N) vs. control (C) group.

Region	Bregma	Fos-Positive Cells	Statistical Outcome
Primary auditory cortex	−3.60, −4.08, −4.56 and −5.04	C: 220 (30) N: 316 (25)	F(1, 16) = 8.897, *p* = 0.009
Medial part of medial geniculate body	−5.04, −5.52, −6.00 and −6.48	C: 455 (29) N: 520 (30)	F(1, 16) = 0.261, *p* = 0.61
Central inferior colliculus	−8.04, −8.52 and −9.00	C: 632 (49) N: 579 (47)	F(1, 16) = 0.088, *p* = 0.77
Dorsal cochlear nucleus	−10.68, −11.16 and −11.64	C: 344 (19) N: 362 (23)	F(1, 16) = 1.075, *p* = 0.31

The results are presented as the mean c-Fos-positive cells/area of each group in combined ipsilateral and contralateral regions ± the SEM. The Bregma level where the readout is taken is mentioned as well.

**Table 3:** C-Fos results of the measured regions in ON vs. OFF status of stimulation.

Region	Bregma	Fos-Positive Cells	Statistical Outcome
Primary auditory cortex	−3.72, −4.08 and −4.44	ON: 189 (11) OFF: 187 (13)	F (1, 18) = 0.010, *p* = 0.92
Central inferior colliculus	−8.04, −8.40 and −8.88	ON: 141 (9) OFF: 121 (17)	F (1, 18) = 2.414, *p* = 0.138
CA1 of hippocampus	−3.36, −3.72 and −4.08	ON: 570 (58) OFF: 668 (124)	F (1, 18) = 0.521, *p* = 0.48
Dentate gyrus	−3.36, −3.72 and −4.08	ON: 206 (20) OFF: 230 (36)	F (1, 18) = 0.338, *p* = 0.568
Basolateral amygdala	−2.64, −3.00 and −3.36	ON: 133 (9) OFF: 116 (10)	F (1, 18) = 1.601, *p* = 0.222
Thalamic reticular nucleus	−3.00, −3.36 and −3.72	ON: 279 (15) OFF: 148 (11)	F (1, 18) = 65.379, *p* < 0.001
Dorsal raphe nucleus	−7.32, −7.68 and −8.04	ON: 117 (28) OFF: 111 (32)	F (1, 9) = 0.029, *p* = 0.869

The results are presented as the mean c-Fos-positive cells/area of each group in combined ipsilateral and contralateral regions ± the SEM. The Bregma level where the readout is taken is mentioned as well.

## Data Availability

Not applicable.

## References

[1] Biswas R., Lugo A., Akeroyd M.A., Schlee W., Gallus S., Hall D.A. (2022). Tinnitus prevalence in Europe: A multi-country cross-sectional population study. Lancet Reg. Health Eur..

[2] Bhatt J.M., Bhattacharyya N., Lin H.W. (2017). Relationships between tinnitus and the prevalence of anxiety and depression. Laryngoscope.

[3] Vanneste S., De Ridder D. (2012). Noninvasive and invasive neuromodulation for the treatment of tinnitus: An overview. Neuromodulation J. Int. Neuromodulation Soc..

[4] Smit J.V., Janssen M.L., Schulze H., Jahanshahi A., Van Overbeeke J.J., Temel Y., Stokroos R.J. (2015). Deep brain stimulation in tinnitus: Current and future perspectives. Brain Res..

[5] Lozano A.M., Lipsman N., Bergman H., Brown P., Chabardes S., Chang J.W., Matthews K., McIntyre C.C., Schlaepfer T.E., Schulder M. (2019). Deep brain stimulation: Current challenges and future directions. Nat. Rev. Neurol..

[6] Luo H., Zhang X., Nation J., Pace E., Lepczyk L., Zhang J. (2012). Tinnitus suppression by electrical stimulation of the rat dorsal cochlear nucleus. Neurosci. Lett..

[7] Smit J.V., Janssen M.L., van Zwieten G., Jahanshahi A., Temel Y., Stokroos R.J. (2016). Deep brain stimulation of the inferior colliculus in the rodent suppresses tinnitus. Brain Res..

[8] Van Zwieten G., Jahanshahi A., van Erp M.L., Temel Y., Stokroos R.J., Janssen M.L.F., Smit J.V. (2019). Alleviation of Tinnitus With High-Frequency Stimulation of the Dorsal Cochlear Nucleus: A Rodent Study. Trends Hear..

[9] Van Zwieten G., Janssen M.L.F., Smit J.V., Janssen A.M.L., Roet M., Jahanshahi A., Stokroos R.J., Temel Y. (2019). Inhibition of Experimental Tinnitus With High Frequency Stimulation of the Rat Medial Geniculate Body. Neuromodulation J. Int. Neuromodulation Soc..

[10] Brinkmann P., Kotz S.A., Smit J.V., Janssen M.L.F., Schwartze M. (2021). Auditory thalamus dysfunction and pathophysiology in tinnitus: A predictive network hypothesis. Brain Struct. Funct..

[11] Koops E.A., Eggermont J.J. (2021). The thalamus and tinnitus: Bridging the gap between animal data and findings in humans. Hear. Res..

[12] Almasabi F., Janssen M.L.F., Devos J., Moerel M., Schwartze M., Kotz S.A., Jahanshahi A., Temel Y., Smit J.V. (2022). The role of the medial geniculate body of the thalamus in the pathophysiology of tinnitus and implications for treatment. Brain Res..

[13] Van Zwieten G., Smit J.V., Jahanshahi A., Temel Y., Stokroos R.J. (2016). Tinnitus: Is there a place for brain stimulation?. Surg. Neurol. Int..

[14] Budzikowski A.S., Vahid-Ansari F., Leenen F.H. (1998). Chronic activation of brain areas by high-sodium diet in Dahl salt-sensitive rats. Am. J. Physiol..

[15] Jahanshahi A., Schonfeld L., Janssen M.L., Hescham S., Kocabicak E., Steinbusch H.W., van Overbeeke J.J., Temel Y. (2013). Electrical stimulation of the motor cortex enhances progenitor cell migration in the adult rat brain. Exp. Brain Res..

[16] Van Zwieten G., Roberts M.J., Schaper F., Smit J.V., Temel Y., Janssen M.L.F. (2021). Noise-induced neurophysiological alterations in the rat medial geniculate body and thalamocortical desynchronization by deep brain stimulation. J. Neurophysiol..

[17] Turner J.G., Brozoski T.J., Bauer C.A., Parrish J.L., Myers K., Hughes L.F., Caspary D.M. (2006). Gap detection deficits in rats with tinnitus: A potential novel screening tool. Behav. Neurosci..

[18] Paxinos G., Watson C. (2006). The Rat Brain in Stereotaxic Coordinates: Hard Cover Edition.

[19] Tan S., Vlamings R., Lim L., Sesia T., Janssen M.L., Steinbusch H.W., Visser-Vandewalle V., Temel Y. (2010). Experimental deep brain stimulation in animal models. Neurosurgery.

[20] Eggermont J.J. (2016). Can Animal Models Contribute to Understanding Tinnitus Heterogeneity in Humans?. Front. Aging Neurosci..

[21] Von der Behrens W. (2014). Animal models of subjective tinnitus. Neural Plast..

[22] Kraus K.S., Canlon B. (2012). Neuronal connectivity and interactions between the auditory and limbic systems. Effects of noise and tinnitus. Hear. Res..

[23] Shore S.E., Roberts L.E., Langguth B. (2016). Maladaptive plasticity in tinnitus--triggers, mechanisms and treatment. Nat. Rev. Neurol..

[24] Shi Y., Burchiel K.J., Anderson V.C., Martin W.H. (2009). Deep brain stimulation effects in patients with tinnitus. Otolaryngol. -Head Neck Surg. Off. J. Am. Acad. Otolaryngol.-Head Neck Surg..

[25] Cheung S.W., Larson P.S. (2010). Tinnitus modulation by deep brain stimulation in locus of caudate neurons (area LC). Neuroscience.

[26] Smit J.V., Janssen M.L., Engelhard M., de Bie R.M., Schuurman P.R., Contarino M.F., Mosch A., Temel Y., Stokroos R.J. (2016). The impact of deep brain stimulation on tinnitus. Surg. Neurol. Int..

[27] Dijkstra E., Figee M., Schuurman P.R., Denys D. (2018). Effective deep brain stimulation of intractable tinnitus: A case study. Brain Stimul..

[28] Cheung S.W., Racine C.A., Henderson-Sabes J., Demopoulos C., Molinaro A.M., Heath S., Nagarajan S.S., Bourne A.L., Rietcheck J.E., Wang S.S. (2019). Phase I trial of caudate deep brain stimulation for treatment-resistant tinnitus. J. Neurosurg..

[29] Muhlau M., Rauschecker J.P., Oestreicher E., Gaser C., Rottinger M., Wohlschlager A.M., Simon F., Etgen T., Conrad B., Sander D. (2006). Structural brain changes in tinnitus. Cerebral Cortex.

[30] Landgrebe M., Langguth B., Rosengarth K., Braun S., Koch A., Kleinjung T., May A., de Ridder D., Hajak G. (2009). Structural brain changes in tinnitus: Grey matter decrease in auditory and non-auditory brain areas. Neuroimage.

[31] Boyen K., Langers D.R., de Kleine E., van Dijk P. (2013). Gray matter in the brain: Differences associated with tinnitus and hearing loss. Hear. Res..

[32] Elgoyhen A.B., Langguth B., De Ridder D., Vanneste S. (2015). Tinnitus: Perspectives from human neuroimaging. Nat. Rev. Neurosci..

[33] Seki S., Eggermont J.J. (2003). Changes in spontaneous firing rate and neural synchrony in cat primary auditory cortex after localized tone-induced hearing loss. Hear. Res..

[34] Weisz N., Dohrmann K., Elbert T., Langguth B., Hajak G., Kleinjung T., Cacace A., Møller A.R. (2007). The relevance of spontaneous activity for the coding of the tinnitus sensation. Progress in Brain Research.

[35] Weisz N., Muller S., Schlee W., Dohrmann K., Hartmann T., Elbert T. (2007). The neural code of auditory phantom perception. J. Neurosci. Off. J. Soc. Neurosci..

[36] Ortmann M., Müller N., Schlee W., Weisz N. (2011). Rapid increases of gamma power in the auditory cortex following noise trauma in humans. Eur. J. Neurosci..

[37] Rauschecker J.P., Leaver A.M., Muhlau M. (2010). Tuning out the noise: Limbic-auditory interactions in tinnitus. Neuron.

[38] De Ridder D., Vanneste S., Langguth B., Llinas R. (2015). Thalamocortical Dysrhythmia: A Theoretical Update in Tinnitus. Front. Neurol..

[39] Kalappa B.I., Brozoski T.J., Turner J.G., Caspary D.M. (2014). Single unit hyperactivity and bursting in the auditory thalamus of awake rats directly correlates with behavioural evidence of tinnitus. J. Physiol..

[40] Cook J.A., Barry K.M., Zimdahl J.W., Leggett K., Mulders W. (2021). Spontaneous firing patterns in the medial geniculate nucleus in a guinea pig model of tinnitus. Hear. Res..

[41] Barry K.M., Robertson D., Mulders W. (2019). Changes in auditory thalamus neural firing patterns after acoustic trauma in rats. Hear. Res..

[42] Ueyama T., Donishi T., Ukai S., Ikeda Y., Hotomi M., Yamanaka N., Shinosaki K., Terada M., Kaneoke Y. (2013). Brain regions responsible for tinnitus distress and loudness: A resting-state FMRI study. PLoS ONE.

[43] Kapolowicz M.R., Thompson L.T. (2016). Acute high-intensity noise induces rapid Arc protein expression but fails to rapidly change GAD expression in amygdala and hippocampus of rats: Effects of treatment with D-cycloserine. Hear. Res..

[44] Zhang L., Wu C., Martel D.T., West M., Sutton M.A., Shore S.E. (2019). Remodeling of cholinergic input to the hippocampus after noise exposure and tinnitus induction in Guinea pigs. Hippocampus.

[45] Zhang L., Wu C., Martel D.T., West M., Sutton M.A., Shore S.E. (2021). Noise Exposure Alters Glutamatergic and GABAergic Synaptic Connectivity in the Hippocampus and Its Relevance to Tinnitus. Neural Plast..

[46] Lanting C.P., de Kleine E., van Dijk P. (2009). Neural activity underlying tinnitus generation: Results from PET and fMRI. Hear. Res..

[47] Adams M.E., Huang T.C., Nagarajan S., Cheung S.W. (2020). Tinnitus Neuroimaging. Otolaryngol. Clin. N. Am..

[48] Qu T., Qi Y., Yu S., Du Z., Wei W., Cai A., Wang J., Nie B., Liu K., Gong S. (2019). Dynamic Changes of Functional Neuronal Activities between the Auditory Pathway and Limbic Systems Contribute to Noise-Induced Tinnitus with a Normal Audiogram. Neuroscience.

[49] Hofmeier B., Wolpert S., Aldamer E.S., Walter M., Thiericke J., Braun C., Zelle D., Rüttiger L., Klose U., Knipper M. (2018). Reduced sound-evoked and resting-state BOLD fMRI connectivity in tinnitus. NeuroImage Clin..

[50] Chen Y.C., Zhang J., Li X.W., Xia W., Feng X., Gao B., Ju S.H., Wang J., Salvi R., Teng G.J. (2014). Aberrant spontaneous brain activity in chronic tinnitus patients revealed by resting-state functional MRI. NeuroImage Clin..

[51] Boyen K., de Kleine E., van Dijk P., Langers D.R. (2014). Tinnitus-related dissociation between cortical and subcortical neural activity in humans with mild to moderate sensorineural hearing loss. Hear. Res..

[52] Carpenter-Thompson J.R., Akrofi K., Schmidt S.A., Dolcos F., Husain F.T. (2014). Alterations of the emotional processing system may underlie preserved rapid reaction time in tinnitus. Brain Res..

[53] Chen Y.C., Li X., Liu L., Wang J., Lu C.Q., Yang M., Jiao Y., Zang F.C., Radziwon K., Chen G.D. (2015). Tinnitus and hyperacusis involve hyperactivity and enhanced connectivity in auditory-limbic-arousal-cerebellar network. eLife.

[54] Ghazaleh N., Zwaag W.V., Clarke S., Ville D.V., Maire R., Saenz M. (2017). High-Resolution fMRI of Auditory Cortical Map Changes in Unilateral Hearing Loss and Tinnitus. Brain Topogr..

[55] Berlot E., Arts R., Smit J., George E., Gulban O.F., Moerel M., Stokroos R., Formisano E., De Martino F. (2020). A 7 Tesla fMRI investigation of human tinnitus percept in cortical and subcortical auditory areas. NeuroImage Clin..

[56] Wallhausser-Franke E., Mahlke C., Oliva R., Braun S., Wenz G., Langner G. (2003). Expression of c-fos in auditory and non-auditory brain regions of the gerbil after manipulations that induce tinnitus. Exp. Brain Res..

[57] Zhang J.S., Kaltenbach J.A., Wang J., Kim S.A. (2003). Fos-like immunoreactivity in auditory and nonauditory brain structures of hamsters previously exposed to intense sound. Exp. Brain Res..

[58] Jia M.H., Qin Z.B. (2006). Expression of c-fos and NR2A in auditory cortex of rats experienced tinnitus. Chin. J. Otorhinolaryngol. Head Neck Surg..

[59] Ouda L., Jilek M., Syka J. (2016). Expression of c-Fos in rat auditory and limbic systems following 22-kHz calls. Behav. Brain Res..

[60] Zhang J., Luo H., Pace E., Li L., Liu B. (2016). Psychophysical and neural correlates of noised-induced tinnitus in animals: Intra- and inter-auditory and non-auditory brain structure studies. Hear. Res..

[61] Basura G.J., Koehler S.D., Shore S.E. (2015). Bimodal stimulus timing-dependent plasticity in primary auditory cortex is altered after noise exposure with and without tinnitus. J. Neurophysiol..

[62] Tan S.K., Janssen M.L., Jahanshahi A., Chouliaras L., Visser-Vandewalle V., Lim L.W., Steinbusch H.W., Sharp T., Temel Y. (2011). High frequency stimulation of the subthalamic nucleus increases c-fos immunoreactivity in the dorsal raphe nucleus and afferent brain regions. J. Psychiatr. Res..

[63] Takata N. (2020). Thalamic reticular nucleus in the thalamocortical loop. Neurosci. Res..

[64] Aizenberg M., Rolon-Martinez S., Pham T., Rao W., Haas J.S., Geffen M.N. (2019). Projection from the Amygdala to the Thalamic Reticular Nucleus Amplifies Cortical Sound Responses. Cell Rep..

[65] Oh K.S., Lee C.J., Gibbs J.W., Coulter D.A. (1995). Postnatal development of GABAA receptor function in somatosensory thalamus and cortex: Whole-cell voltage-clamp recordings in acutely isolated rat neurons. J. Neurosci. Off. J. Soc. Neurosci..

